# Candidate Cyanide Resistance Genes in Eutardigrade (Tardigrada) Genomes and KCN Resistance of *Hypsibius exemplaris*

**DOI:** 10.3390/ijms27114946

**Published:** 2026-05-29

**Authors:** Tomasz Bartylak, Monika Mioduchowska, Hanna Kmita, Łukasz Kaczmarek

**Affiliations:** 1 Department of Animal Taxonomy and Ecology, Faculty of Biology, Adam Mickiewicz University in Poznań, Uniwersytetu Poznańskiego 6, 61-614 Poznań, Poland; kaczmar@amu.edu.pl; 2 Department of Bioenergetics, Faculty of Biology, Adam Mickiewicz University in Poznań, Uniwersytetu Poznańskiego 6, 61-614 Poznań, Poland; hanna.kmita@amu.edu.pl; 3 Department of Evolutionary Genetics and Biosystematics, Faculty of Biology, University of Gdańsk, Wita Stwosza 59, 80-308 Gdańsk, Poland

**Keywords:** extremotolerance, cyanide-resistant respiration, mitochondrial alternative oxidase, rhodanese, nitrilase, detoxification pathways

## Abstract

Several eutardigrade species can withstand high doses of potassium cyanide (KCN), a potent inhibitor of mitochondrial respiration, suggesting defense mechanisms that remain poorly understood. We surveyed the available tardigrade genomes in search for 11 protein families associated with cyanide detoxification and resistance in other organisms. We identified 28 sequences putatively encoding mitochondrial alternative oxidase (AOX), as well as proteins from the nitrilase superfamily and rhodanese superfamily in three eutardigrades, but found no evidence for other canonical cyanide-detoxifying enzymes such as β-cyanoalanine synthase. We also experimentally tested KCN resistance of the model tardigrade species *Hypsibius exemplaris* using a standard exposure protocol (100 mM KCN, 10 min) to determine if its poor anhydrobiotic ability reflects on its capacity for KCN resistance. All specimens survived KCN exposure, with a mean recovery time that did not differ significantly from other tardigrade species, previously tested under identical conditions, suggesting that KCN resistance may be a shared trait among eutardigrades. However, confirming whether this represents a conserved feature across the Eutardigrada class will require broader sampling. While these findings highlight potential pathways, they are limited by the lack of functional or expression validation for the identified genes.

## 1. Introduction

The phylum Tardigrada consists of over 1500 species [1] of microscopic invertebrates [2,3]. They are renowned for their extensive ability to resist various kinds of environmental stressors, including amongst others freezing, osmotic stress, radiation, toxic chemical compounds and desiccation [4,5,6,7,8,9]. This ability is generally expressed through cryptobiosis—a reversible state of suspended animation in which the organism’s metabolism slows to a near-undetectable level [10,11]. Several types of cryptobiosis are commonly delineated, based on stress factors that induce them [2]. These include: anhydrobiosis (induced by desiccation), cryobiosis (induced by freezing), osmobiosis (induced by osmotic stress), anoxybiosis (induced by lack of oxygen) and chemobiosis (induced by toxic chemical substances) [2]. In recent years, however, anoxybiosis has started to be questioned as a legitimate type of cryptobiosis, mainly on basis of lack of evidence of reversible cessation of metabolism and lack of morphological changes associated with other types of cryptobiosis [12]. While their ability to cope with lack of water (anhydrobiosis) is relatively well studied and well understood [11,13,14,15], other types of stress resistance exhibited by these animals, particularly their ability to cope with toxic chemical agents, remain comparatively obscure [5].

Potassium cyanide (KCN) is a water-soluble toxin [16]. It rapidly converts to hydrogen cyanide (HCN) [17]. While the free cyanide anion (CN^−^) itself cannot easily penetrate through the lipid bilayer, HCN dissolves in fats and is able to easily diffuse across cell membranes. It is highly toxic, with a lethal dose for humans estimated to be between 1 and 2 mg/kg ingested orally [18]. The KCN owes its toxicity to the ability of cyanide anions to bind to the ferric iron (Fe^3+^) found within complex IV (cytochrome c oxidase) of the mitochondrial respiratory chain (MRC). This binding prevents the sequential flow of electrons, stopping the transfer of electrons from cytochrome c to molecular oxygen [19]. This causes blockages in electron transport through the MRC, halting respiration and preventing ATP production, which results in cell death [17]. This is particularly deadly to cells and tissues with high energy demands [19]. While cessation of respiration is the most dangerous, it is not the only danger posed by cyanide to living cells. Cyanide-induced interruption of the MRC, specifically the accumulation of electrons upstream, can lead to the generation of reactive oxygen species (ROS) [19], which in turn inflict significant oxidative stress, even if the affected organism survives initial hypoxia.

There exist numerous mechanisms enabling living organisms to resist the toxic effect of cyanide (Table 1). Even a single organism may exhibit more than one pathway depending on external conditions such as pH, oxygen, and cyanide concentration [20]. These mechanisms of resistance can be divided into four distinct groups: enzymatic pathways relying on substitution and transfer, hydrolytic and oxidative reactions, and cyanide-resistant respiration [21].

### 1.1. Substitution/Transfer Pathways

Substitution and transfer pathways involve enzymatic reactions that neutralize cyanide by converting it into significantly less toxic derivatives, such as thiocyanate or β-cyanoalanine [21,24]. The most prevalent enzyme in this group is rhodanese (Table 1), which is found in nearly all living organisms [22,23] and supports cellular sulfur homeostasis and redox balance in addition to its detoxification role. Other key pathways utilize β-cyanoalanine synthase (β-CAS; Table 1), which is primarily found in plants and certain arthropods [24,32], or 3-mercaptopyruvate sulfurtransferase (3-MPST; Table 1), an enzyme evolutionarily related to rhodanese [22,33]. Additionally, enzymes such as cystathionine λ-lyase (CST; Table 1) provide indirect support by generating the sulfur-containing substrates necessary for these clearance mechanisms [26,34].

### 1.2. Hydrolytic Reactions

Hydrolytic pathways degrade nitriles and cyanide compounds by cleaving carbon–nitrogen bonds to form less toxic products such as carboxylic acids, amides, or ammonia [25,35]. Nitrilases (Table 1) represent a widely distributed superfamily of enzymes that can directly hydrolyze nitriles to their corresponding acids [36] and also detoxify β-cyanoalanine produced by β-CAS pathways [36,37]. In some invertebrates, including *Drosophila melanogaster melanogaster* Meigen, 1830 [38] and *Caenorhabditis elegans* Maupas, 1900 [39], nitrilase exists as a fusion protein (NitFhit; Table 1) containing both a nitrilase domain and a fragile histidine triad (Fhit) domain [27,40]. Other hydrolytic enzymes, such as cyanide hydratases, nitrile hydratases, and cyanidases (Table 1), are primarily restricted to fungi and bacteria [20,25,40].

### 1.3. Oxidative Reactions

Cyanide oxygenases encompass two distinct classes of enzymes. The first one is cyanide monooxygenase (Table 1), which converts cyanide to cyanate [21,25], which is then converted to carbon dioxide and ammonia by another enzyme, cyanase [20,25]. The second one is cyanide dioxygenase (Table 1), which directly oxidizes cyanide to carbon dioxide and ammonia, without an intermediate cyanate or involvement of cyanase [20,25]. Both enzyme groups are primarily found in microorganisms like bacteria and fungi [20,21,25].

### 1.4. Cyanide-Resistant Respiration

In contrast to previously discussed mechanisms of cyanide detoxification, which rely on removing the toxin outright before it can cause damage to the cells, mechanisms based on alternative respiration seek instead to prevent the damage from occurring, by providing effective ways to bypass the cyanide-susceptible components of the MRC. Isolated mitochondria, as well as whole animals of two millipede species, have been found to withstand exposure to cyanide [41]. This ability was attributed to either a large excess of normal cytochrome oxidase or presumed existence of a resistant terminal oxidase [41].

**Figure 1:**
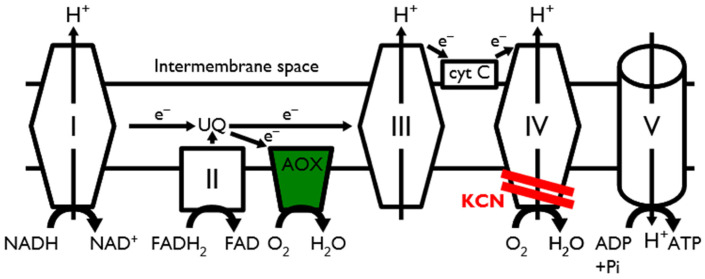
Scheme of the mitochondrial respiratory chain. Complexes I, III and IV transport protons into the intermembrane space, forming a proton gradient, which is then used by complex V for ATP synthesis. Cyanide-induced inhibition of complex IV indicated in red, alternative oxidase (AOX) indicated in green.

Another mechanism is mitochondrial alternative oxidase (AOX), which forms a bypass that transfers electrons directly from the ubiquinone pool to molecular oxygen (Figure 1), thereby circumventing MRC complexes III and IV, the latter being a target for cyanide toxicity [25,28,29,30,42]. Because AOX bypasses the proton-pumping sites of complexes III and IV, it contributes to thermogenesis [43]. The physiological role of AOX is also to maintain the electron flow under stress conditions that impair the conventional MRC and to limit the resulting overproduction of reactive oxygen species (ROS) that results from the over-reduction of the ubiquinone pool [28,31]. A functional AOX has been found in plants, fungi, protists, and several groups of animals, including tardigrades [28,30,44], and it has been suspected that AOX plays a part in tardigrade anhydrobiosis [42], particularly during long-term desiccation periods or when combating associated oxidative stress [15,42].

In recent years, two tardigrade species have been shown to withstand the toxic effects of potassium cyanide (KCN), fully recovering after a 10 min treatment with 100 mM KCN followed by transfer to clean culture medium [45,46]. These are *Paramacrobiotus experimentalis* Kaczmarek, Mioduchowska, Poprawa & Roszkowska, 2020 [47] and *Macrobiotus naginae* (Vecchi, Stec, Vuori, Ryndov, Chartrain & Calhim, 2022) [48]. Notably, at no point between exposure and eventual recovery do these tardigrades exhibit tun formation or any other obvious morphological changes beyond simple straightening of the body [45], which are considered typically associated with tardigrade anoxybiosis [3,5,49]. They also do not require preconditioning to achieve survival under these conditions [45].

Another species tested for KCN resistance was *Hypsibius exemplaris* Gąsiorek, Stec, Morek & Michalczyk, 2018 [50]. However, while *Pam. experimentalis* and *Mac. naginae* were tested with concentrations ranging from 100 to 300 mM KCN [46], *Hys. exemplaris* was only treated with a comparatively negligible 1 mM, using a protocol highly divergent from the one employed for the other two species (i.e., tardigrades were placed into experimental vessel first and then 500 mM KCN was added, resulting in final solution of 1 mM, potentially briefly exposing tardigrades to undiluted KCN solution; they were not transferred into clean water but remained in the solution, and their time to recovery was not measured) [30]. Therefore, while it is confirmed that it possesses some degree of KCN resistance, its extent in comparison to other tardigrades has remained unclear so far. Using respiration measurement, estimation of the mitochondrial inner membrane potential and mass spectrometry, the study determined that *Hys. exemplaris* possesses an active AOX [30]. The same study also suggests that cyanide anions may influence AOX expression in tardigrade cells [30].

Currently, AOX-mediated respiration is therefore suggested to be a key mechanism underlying cyanide resistance in tardigrades [30]. However, it remains unclear whether it is a sole factor or a part of a larger, still not fully understood system. No other mechanisms of cyanide resistance have been identified in tardigrades as of today.

Tardigrades also stand out amongst other invertebrates exhibiting cyanide resistance. Most of the animal species recorded as possessing such quality experience regular contact with the poison, either a part of their diet (i.e., multiple species of moths) [24,51,52,53] or as a defense mechanisms (i.e., certain species of millipedes) [41]. In contrast, tardigrades neither exhibit a cyanide-rich diet, nor use it to avoid predation.

*Hypsibius exemplaris* is a freshwater tardigrade belonging to the family Hypsibiidae. It is parthenogenetic, laying eggs in exuviae [54], and herbivorous, feeding primarily on algae [50]. It is characterized as being easy to culture and possesses a relatively short generation time (specimens can start producing eggs eight days after hatching) [54], making it suitable for use in laboratory conditions. It has a body length ranging between 100 and 300 µm [50], making it comparatively small for an eutardigrade. As of today, it is also one of the few tardigrades whose genome had been successfully sequenced [55]. Owing to its name as an “exemplar”, it is one of the most widely used model tardigrade species [56], utilized in studies concerning evolution [57,58,59], extremotolerance [30,60,61], genetics [62,63,64] and a wide variety of other areas of research [65,66,67,68]. In recent years, however, the extent of its capacity for anhydrobiosis has been put into doubt, exhibiting low recovery rates when compared to other tardigrade species [6,69]. Its aquatic natural habitat, small body size, low anhydrobiotic abilities and ubiquitous status in tardigrade research together make *Hys. exemplaris* a good point of comparison with two species previously tested for KCN resistance [46].

In this study we pursued three distinct objectives. First, we identified candidate genes encoding homologs of known cyanide defense proteins through a genomic survey of available tardigrade genome assemblies, documenting their presence/absence and distribution across species. Second, we experimentally confirmed KCN tolerance in *Hys. exemplaris*, a common model tardigrade, using a unified exposure protocol (100 mM KCN, 10 min), and compared the results with analogous experiments conducted using other eutardigrade species, to assess whether KCN resistance is conserved across eutardigrades or restricted to species with robust anhydrobiotic capacity. Third, we discussed mechanistic hypotheses suggested by the overall pattern of gene presence and phenotypic results, particularly the potential role of mitochondrial alternative oxidase. While remaining speculative and requiring functional validation, our results help outline the direction for future studies. Abbreviations of tardigrade genera names follow Perry et al. [70].

## 2. Results

### 2.1. Tardigrades Possess Genes Encoding Some Proteins Associated with Potassium Cyanide (KCN) Resistance

In total, 28 genes encoding proteins, presented in Table 1, were identified in the available genomic data of three eutardigrade species: *Hys. exemplaris*, *Paramacrobiotus metropolitanus* Sugiura, Matsumoto & Kunieda, 2022 [71] and *Ramazzottius varieornatus* Bertolani & Kinchin, 1993 [72] (Table 2). The genome assemblies are available in GenBank under the accession numbers GCA_002082055.1, GCF_019649055.1 and GCA_001949185.1 respectively. Among them, AOX, beta-ureidopropionase, NitFhit, and pantetheinase were identified in all three analyzed tardigrade genomes. *Hypsibius exemplaris* was the only species in which a gene encoding a carbon–nitrogen hydrolase was detected. Several rhodanese-like proteins were identified in *Hys. exemplaris* and *Pam. metropolitanus* but were not found in *Ram. varieornatus*. Instead, *Ram. varieornatus* was found to possess a 3-MPST sequence and was the only examined species in which this sequence was detected (Table 2).

Homology assignment was supported using BLAST v. 2.17.0 searches and analyses of amino acid sequence identity. In addition, phylogenetic reconstruction revealed that the identified homologs clustered into distinct clades corresponding to the nitrilase superfamily and the rhodanese superfamily, with the majority of branches supported by high bootstrap values (reaching 100) (Figures S1 and S2, Tables S1 and S2).

All identified proteins contained conserved domains corresponding to their respective protein families: the rhodanese-like domain (PFAM_ID: PF00581) was detected in all rhodanese superfamily sequences (Figure S2); the vanin C-terminal (PFAM_ID: PF19018), carbon–nitrogen hydrolase (PFAM_ID: PF00795), and HIT domains (PFAM_ID: PF01230) were identified in the nitrilase superfamily sequences (for details on domain distribution in the analyzed sequences, see Figure S1); and the alternative oxidase domain (PFAM_ID: PF01786) was found in the alternative oxidase sequences. These annotations are based on conserved sequence motifs and homology to characterized proteins; however, functional assignments remain putative. Structural validation is required to confirm catalytic activity and to distinguish between active enzymes and potentially non-functional homologs.

### 2.2. Hypsibius exemplaris Exhibits Resistance to KCN Comparable to Other Tardigrade Species

**Figure 2:**
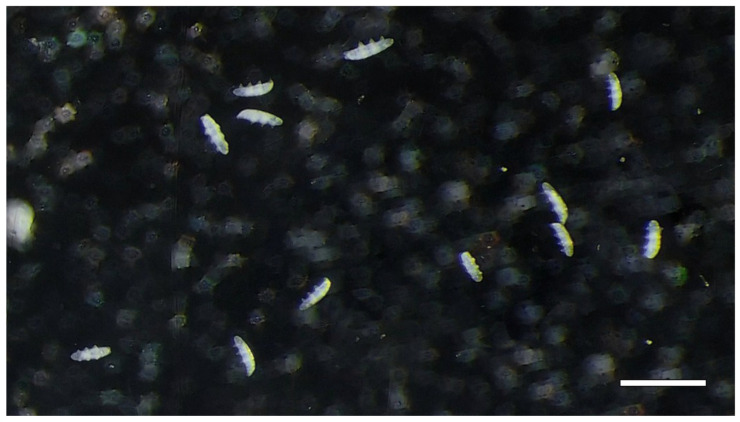
Specimens of *Hys. exemplaris* immobilized following a 10 min exposure to 100 mM potassium cyanide (KCN). Scale bar represents 500 μm.

In every replicate, tardigrades immediately and completely stopped their movement (Figure 2). Following transfer to clean culture medium, all specimens eventually recovered and returned to full activity. All control specimens remained active throughout the observation period.

Mean recovery time is presented in Table 3. When compared with mean recovery time of *Pam. experimentalis* and *Mac. naginae* treated with 100 mM KCN, taken from Bartylak et al. [46], no statistically significant differences were found among species (F(2, 87) = 0.1801, *p* = 0.8355). Replicates were consistent within each species, with no variance attributable to differences among them (SD = 0.000); all variation occurred within replicates (SD = 6.571 min).

## 3. Discussion

### 3.1. Genomic Survey and KCN Resistance

Comparative analysis of sequences identified in the genomes of *Hys. exemplaris*, *Pam. metropolitanus*, and *Ram. varieornatus* revealed a shared set of conserved proteins, as well as species-specific differences in gene content (Table 4). Alternative oxidase (AOX) and several members of the nitrilase superfamily, including beta-ureidopropionase (UPB1), NitFhit proteins, and pantetheinase, were detected in all three species (Table 4). Differences were observed in the number of NitFhit sequences, with *Pam. metropolitanus* exhibiting the highest number of homologs. A carbon–nitrogen hydrolase was identified exclusively in *Hys. exemplaris* (Table 4). The most notable variation concerned proteins of the rhodanese superfamily: multiple rhodanese-like sequences were present in *Hys. exemplaris* and *Pam. metropolitanus*, whereas no canonical rhodanese proteins were detected in *Ram. varieornatus*, which instead encoded a single 3-mercaptopyruvate sulfurtransferase (3-MPST) containing two rhodanese domains (Table 4).

Rhodanese is probably the most widely distributed enzyme associated with cyanide detoxification, both amongst arthropods and animals in general, whether they are regularly exposed to the toxin or not [51], and therefore its presence in tardigrades is not entirely surprising. However, its primary role in arthropods remains somewhat debated. Aside from cyanide detoxification, rhodanese plays a role in other metabolic pathways, including the regulation of cellular sulfur homeostasis, iron–sulfur cluster formation and maintaining redox balance [22,23,24]. A study involving measuring its activity in Pierinae butterflies showed no correlation between consumption of cyanogenic plants and rhodanese activity [75]. Its authors suggested that cyanide detoxification may not be the primary role of rhodanese in insects [75]. In general, rhodanese activity in invertebrates is suggested to be lower than that of vertebrates [51]. Therefore, while discovery of rhodanese in tardigrades potentially provides additional insight into their ability to resist KCN exposure, its role cannot be confirmed without measuring its activity. Importantly, rhodanese contributes to a wide array of cellular processes [22] and the mere presence of rhodanese-encoding sequences does not establish its involvement in the observed KCN resistance.

One of the analyzed species, *Ram. varieornatus*, showed no presence of rhodanese in its genome. It did however possess 3-MPST (Table 2), which also belongs to rhodanese superfamily and is characterized by its two rhodanese domains [25,33]. This is somewhat surprising, considering that both *Pam. metropolitanus* and *Hys. exemplaris* showed multiple sequences of rhodanese-like proteins.

The nitrilase superfamily is also widely distributed, being found in plants, fungi, protozoa and animals [20,25,35,37]. In analyzed tardigrade genomes, genes encoding several proteins from this superfamily were found. However, the identified eutardigrade sequences belong to subfamilies (NitFhit, beta-ureidopropionase, pantetheinase, carbon–nitrogen hydrolase) whose primary functions are unrelated to cyanide detoxification. While nitrilases in a strict sense can contribute to cyanide detoxification in tandem with β-CAS by hydrolysing β-cyanoalanine [76,77], no β-CAS genes were identified in any of the analyzed tardigrade genomes, making this pathway unavailable. The absence of β-CAS may reflect a broader phylogenetic distribution of this enzyme rather than species-specific adaptation.

As already mentioned, what is also interesting is that none of the identified genes corresponded to β-CAS, which is a cyanide detoxification protein, found within a variety of arthropod groups, including Acari, Diptera Lepidoptera and Orthoptera [24]. However, its distribution in arthropods is patchy, with phylogenetic analyses indicating that its origins may lay in horizontal gene transfer from bacteria [78], with different lineages acquiring it independently, rather than inheriting it from a common ancestor [78]. It should also be noted that many of the species possessing β-CAS activity are exposed to cyanide via consumed food [78]. Tardigrades meanwhile are not known to consume cyanide-rich food.

The absence of genes encoding other selected enzymes in the studied tardigrade genomes (cyanide hydratase, nitrile hydratase, cyanidase, cyanide oxygenase, cyanase, β-cyano-α-aminobutyric acid synthase, cystathionine λ-lyase) is less surprising as they are primarily be found in bacteria, as well as some fungi and mammals, while generally being absent in invertebrates, with the exception of certain parasitic helminths [22,25,26,75,78,79,80]. Their absence is therefore in line with current state of knowledge, which holds that horizontal gene transfer is not a hallmark of tardigrade genomes [81].

Previous research concerning the effects of KCN on *Hys. exemplaris* treated it with 1 mM KCN [30]. Based on results obtained for other eutardigrades, which were able to easily cope with 100 times higher concentrations [45,46], it was suspected that even if *Hys. exemplaris*’s resistance to KCN is comparatively lesser, it should still be able to withstand higher doses. Given its persistent status as a model organism for tardigrades [60,61,62,64,66], it was vital to provide up-to-date data for it in this area of study.

Results presented in the current study confirm that suspicion, having subjected specimens of *Hys. exemplaris* to 100 mM KCN. Not only was it found that all specimens survived the exposure and were able to successfully recover to full activity, but their mean time of recovery was not found to be significantly different from that of *Pam. experimentalis* and *Mac. naginae*, tested in previous studies [46].

Testing of *Hys. exemplaris* was particularly important in the light of the current study as it is the only tardigrade species tested for KCN resistance so far that also has its complete genome available. This made it possible to directly link any mechanisms identified in genome data with in vivo confirmation of KCN resistance in this tardigrade species.

Moreover, *Hys. exemplaris* is an aquatic species while the other species tested, *Pam. experimentalis* and *Mac. naginae*, are respectively limnoterrestrial and xerophilic [46]. It has been suggested that there may exist a link between tardigrade KCN resistance and anhydrobiotic capacity [15,42,45]. A relationship between anhydrobiotic ability and occupied habitat has also been observed, with those most likely to encounter draught (i.e., terrestrial species) generally exhibiting better capacity for anhydrobiosis, when compared to aquatic species [6,69,82,83]. In light of this knowledge, it was important to see if this relationship would transfer over to KCN resistance. Moreover, *Hys. exemplaris* has been noted as having poor anhydrobiotic capacity compared to other eutardigrades [69]; thus, testing whether this species possesses comparable KCN resistance to other species with better anhydrobiosis performance would help determine if cyanide tolerance correlates with cryptobiotic ability or represents a broadly conserved trait across Eutardigrada. Obtained results show that for 100 mM KCN, 10 min exposure, this is not the case. This may suggest that while some mechanisms responsible for anhydrobiosis aid in response to KCN treatment (particularly the AOX [30,42]), not all of them play a role in that process.

This hypothesis may support the claim that a base level of KCN resistance is widespread amongst eutardigrades, regardless of their diet, habitat, mode of reproduction or degree of anhydrobiotic capacity. Confirming this will require broader sampling, spanning the wide breadth of the Eutardigrada. While the extent of KCN resistance might differ between individual species and genera, such differences are not detectable after 10 min treatment with 100 mM KCN.

Considering current findings, there are no explicit clues indicating that tardigrades produce any currently known proteins or enzymes capable of active detoxification of cyanide within their cells. While this does not completely rule out the possibility of detoxification mechanisms of a different nature existing in tardigrades, our genomic survey did not identify evidence for them. Instead, their strategy may involve minimizing damage and weathering the toxic environment, with the help of AOX-enabled, cyanide-resistant respiration and then recovering once stress factor is removed. This is consistent with a previous study by Wojciechowska et al. [30] which established that *Hys. exemplaris* specimens exposed to KCN partially recovered over 2 h, with recovery abolished by the AOX inhibitor BHAM, strongly indicating that functional AOX contributes to cyanide tolerance in tardigrades. However, further validation through enzyme activity assays or expression profiling during KCN exposure in multiple species would be necessary to firmly establish this across eutardigrades as a whole.

This aligns well with previous study, which showed that exposure time is one of the most important factors affecting recovery time and ultrastructural changes occurring in tardigrades because of KCN treatment [45].

It should also be kept in mind that tardigrades are unlikely to encounter the concentrations of KCN as high as those tested here in their natural habitats [53]. Therefore, it is almost certain that their mechanisms of toxin resistance primarily serve another purpose and make up only a part of larger system of cellular defense. AOX has already been linked to tardigrade anhydrobiosis [42] and it is possible that other proteins associated with anhydrobiosis or other forms of cryptobiosis contribute to cyanide resistance and subsequent recovery [15].

### 3.2. Future Perspectives

Another angle meriting consideration is microbiome. The host-associated microbiome contributes to host fitness by enhancing nutrient acquisition, facilitating metabolism of otherwise inaccessible compounds and regulating immune system development and defense against pathogens [84]. This mutualistic interaction can modulate immune responses, promote host development and improve tolerance to environmental stressors, making microbiome–host interactions a key component of host physiology and ecological adaptation [85].

In our previous study, microbiome profiling was performed across various developmental and physiological stages of two *Paramacrobiotus* species, including: eggs; active adult specimens (both before and after 7 and 120 days of anhydrobiosis, referred to as short- and long-term anhydrobiosis, respectively); specimens in the desiccated tun stage; and dead specimens following long-term anhydrobiosis (no dead specimens were observed after short-term anhydrobiosis). It was shown that the microbiome community varied among stages, with high stage specificity [86]. This stage specificity suggests that some bacteria could play a functional role in supporting stress responses, either by producing metabolites that enhance cellular protection or by modulating host metabolic pathways [87,88]. Moreover, functional predictions (based on microbial gene content) revealed increased levels of trehalose synthase and heat shock protein genes during environmental stress [86].

However, it should be emphasized that the present study does not directly assess the role of the microbiome in cyanide detoxification, and the considerations below are therefore speculative and based on indirect evidence and previous literature. The resistance to cyanide exposure could also possibly be supported by products of the microbiome community within tardigrades. Species of bacteria from various genera, including *Pseudomonas* Migula 1894 [89], *Bacillus* Cohn 1872 [90], and *Acinetobacter* Brisou & Prévot 1954 [91], were detected in tardigrades from genera *Hypsibius* Ehrenberg 1848 [92] and *Paramacrobiotus* [47,66,93,94]. These bacteria possess enzymes capable of cyanide detoxification, including rhodanese-like sulfurtransferases, nitrilases, or cyanide-insensitive oxidases [21,25,40]. While these taxa are mostly environmentally acquired and transient, their metabolic potential raises the possibility that they could aid in cyanide detoxification. Importantly, no functional assays or direct measurements performed in this study confirm such activity in the analyzed systems. Taken together, these findings could suggest a nuanced role of the tardigrade microbiome in chemical stress responses. In anhydrobiosis, certain microbial taxa may enhance the host resilience by supporting protective pathways or stabilizing metabolites [95,96]. This interpretation should be treated as a working hypothesis rather than a conclusion derived from the present data. We hypothesize that during cyanide exposure, the microbiome may function as a passive metabolic filter, without replacing the intrinsic detoxification strategies of the host. In both cases, the extraordinary resistance of tardigrades is fundamentally rooted in their own physiological adaptations (such as reversible metabolic suppression, protective molecule synthesis, and structural stabilization [15]), while the microbiome may provide supplementary support. Some bacteria are known to interact with cyanide not only by enzymatic degradation, but also through physical sequestration mechanisms [97,98]. For example, certain microbial cells can bind cyanide at the cell surface and accumulate it within extracellular matrices or biofilms, altering local diffusion conditions and potentially reducing the effective concentration of free cyanide in their environment [99]. In addition, microbial metabolism can convert cyanide into less reactive derivatives such as thiocyanate via sulfurtransferase pathways, which has been documented as a detoxification route in bacterial systems [21]. These processes would effectively act as a biological filter, lowering local free cyanide levels and mitigating toxicity [97]. Nevertheless, such mechanisms have not been demonstrated in tardigrade-associated microbiomes and remain to be experimentally verified.

Considering the relationship between the host and its microbiome may inspire new experiments to explore its role in tolerance to extreme environments. One approach could involve comparing the survival and recovery of organisms with an intact microbiome to those whose microbiome has been artificially depleted. Such experiments would be necessary to directly test the hypotheses proposed above. Investigating the potential contribution of the microbiome to cyanide resistance represents a key direction for future research. Moreover, our results further support the previously proposed hypothesis that alternative oxidase (AOX) is a primary mechanism underlying cyanide resistance in tardigrades. However, to confirm these findings, additional experimental validation is necessary, including testing the effects of AOX inhibitors such as BHAM [44] on tardigrade KCN resistance. Subsequent studies could combine KCN exposure with transcriptomic and proteomic profiling to determine whether the expression of the proteins identified here is induced by cyanide. Beyond multi-omic approaches, pharmacological inhibition of AOX and other candidate proteins could help clarify their specific roles in the tardigrade response to KCN treatment.

Overall, this study provides a valuable foundation for future investigations by highlighting potential genes and proteins of interest and emphasizing the need for integrative, microbiome-focused analyses to assess whether microbial communities may indirectly contribute to toxin resistance.

## 4. Materials and Methods

### 4.1. Protein List Construction and Database Search

Based on the surveyed literature, 11 proteins were chosen as candidates for involvement in mechanisms of defense against cyanide toxicity (Table 1). These genes were searched in all available tardigrade genomes using a combined homology-based and manual validation approach. Initial candidate sequences were retrieved from tardigrade genomes using keyword-based searches and sequence retrieval in the NCBI Sequence Set Browser. Downloaded sequences were then subsequently subjected to quality control and filtering prior to downstream analyses.

Low-complexity regions were filtered using SEG implemented in BLASTP v. 2.17.0, as low-complexity and compositionally biased regions are known to generate false matches during homology searches [100]. Sequences containing extensive low-complexity regions (>40% masked residues) or unusually short protein sequences (<100 amino acids) were excluded from further analyses. To identify potential contaminant sequences (bacterial or fungal sequences lacking detectable eukaryotic homologs), all remaining candidates were compared against NCBI non-redundant (nr) and Swiss-Prot databases using BLASTP v. 2.17.0 [101]. BLAST searches implemented in Geneious Prime v.2026.0.2 [102] were additionally used to ensure high-confidence detection of homologs corresponding to the same protein and stringent filtering criteria were applied. Specifically, hits were retained only if they met an E-value threshold of ≤1 × 10^−30^, a minimum sequence identity of ≥97%, and a minimum alignment coverage of ≥90% for both query and subject sequences. These conservative parameters were selected to minimize the inclusion of distant homologs or partial matches and to ensure that only closely related protein sequences were considered.

Following automated filtering, a manual validation of candidate sequences was applied to ambiguous cases. This included verification of alignment quality, absence of stop codons (for coding regions), and consistency of taxonomic assignment across top BLAST hits. Manual decisions were guided by the same predefined criteria to ensure consistency and reproducibility.

To confirm homology and assign the sequences to known enzyme families, amino acid sequences of the target proteins identified as belonging to the nitrilase superfamily and rhodanese superfamily were aligned using Clustal Omega v. 1.2.4 and phylogenetic dendrograms were constructed using the Neighbor-Joining method with 1000 bootstrap replicates in the Geneious Tree Builder (implemented in Geneious Prime v. 2026.0.2) using default settings. Pairwise distances, expressed as percentage sequence identity among all analyzed amino acid sequences, were also calculated in Geneious Prime.

Classification of protein families and their associated domains was performed using the InterPro 108.0. resource (available at EMBL-EBI https://www.ebi.ac.uk/interpro/ (accessed on 30 April 2026) [103]. Domain annotations were further verified based on assignments from the Pfam database (Pfam 35.0) [104] to ensure consistency and reliability of the predicted functional features.

Figure 1 was created using Inkscape 1.4.

### 4.2. Hypsibius exemplaris Culture

The specimens of *Hys. exemplaris* used in the study were obtained through lab culture using a modified version of a protocol presented by Roszkowska et al. [105]. Tardigrades from the Z151 strain (Sciento, Manchester, UK) were kept in plastic Petri dishes, with bottoms scratched using sandpaper, filled with culture medium consisting of a 1:3 mixture of double-distilled water and ŻYWIEC ZDRÓJ mineral water (ŻYWIEC ZDRÓJ, Warsaw, Poland). Culture dishes were kept in a POL EKO KK 115 TOP+ climate chamber (POL EKO, Wodzisław Śląski, Poland) with photoperiod 24 h light, 20 °C, relative humidity (RH) 50%. Once a week, the culture medium was replaced with a fresh one and tardigrades were fed with algae *Chlorella vulgaris* Beijerinck 1890 [106]. Adult specimens, fully active (displaying coordinated movements of the body and legs) and non-molting, with body length ranging from 240 to 260 μm were selected for use in experiments.

### 4.3. KCN Exposure Protocol

Treatment of tardigrades with KCN followed a protocol established and refined in previous studies [45,46] that is in all regards identical to that used by Bartylak et al. [46]. Tardigrades were placed in groups of 10 specimens in glass staining blocks containing 2 mL of 100 mM KCN solution in culture medium (pH = 7.2–7.4). They were treated for 10 min and then flushed by moving them two times into glass staining blocks containing 2 mL of culture medium to remove remaining KCN. Tardigrades were observed under an Olympus SZX7 stereomicroscope (Olympus Corporation, Shinjuku-ku, Japan), connected to a Techrebal Orange PRO 4 K camera (Techrebal, Wilczyce, Poland). Observation was continued for three hours. The number of active specimens was noted every five minutes. The activity was defined as visible, coordinated movements of legs, following Roszkowska et al. [6]. If by the end of the observation period there were still any non-active animals, the group was left overnight in culture conditions and observed again after 24 h to confirm whether the remaining specimens had recovered. Transfer of tardigrades from KCN solution to observation vessel took ca. 30 s and was not considered when analyzing obtained results. Tardigrades used in experiments were only treated once and following the experiment were disposed of. Three repetitions each consisting of 10 specimens were conducted. The control group consisted of specimens extracted from the culture and not treated with KCN and was observed in the same way as the treated group.

Figure 2 was created from a photo captured using the experimental setup. As the specimens were dispersed across a large field of view in the original image, individual tardigrades were repositioned using Adobe Photoshop v. 21.0.2 (Adobe, San Jose, CA, USA) to improve visibility and readability of the figure. Out-of-focus dust particles and debris were also removed.

### 4.4. Statistical Analysis

Mean values of time needed for tardigrades to recover were calculated and presented in a table, alongside with standard deviations. To assess the ability of *Hys. exemplaris* to withstand exposure to KCN in relation to other tardigrade species, collected data was compared to results of *Pam. experimentalis* and *Mac. naginae* subjected to identical KCN concentration and exposure time, referenced from previously conducted studies using the exact same experimental protocol [46]. To compare the differences in recovery time and to address potential non-independence of the results, a nested one-way ANOVA was performed [107]. Prior to analysis, normality was assessed within each group using four tests: Anderson–Darling, D’Agostino and Pearson, Shapiro–Wilk, and Kolmogorov–Smirnov. Three of the four tests indicated mild departures from normality in two replicates of *Hys. exemplaris*. Given the balanced design and the well-documented robustness of the F-test to minor normality violations [108,109], parametric analysis was considered appropriate. All analyses were performed in GraphPad Prism 8 (GraphPad Software, San Diego, CA, USA).

## Figures and Tables

**Table 1:** Proteins selected for identification in tardigrade genomes available in genetic databases, with summary of their mechanism and organisms possessing them.

Protein Name	Mechanism	Organisms	Source
Substitution and transfer pathways			
rhodanase	Transfers sulfur from thiosulfate to cyanide via a double displacement reaction, forming sulfite and thiocyanate	Almost all living organisms from bacteria to humans	[22,23]
β-cyanoalanine synthase	Substitutes cysteine’s sulfhydryl group with cyanide, forming β-cyanoalanine and hydrogen sulfide	Plants, bacteria, certain arthropods	[24,25]
γ-cyano-α-aminobutyric acid synthase	Alternative cyanide assimilation pathway synthesizing γ-cyano-α-aminobutyric acid, a β-cyanoalanine homolog	Bacteria	[24,25]
3-mercaptopyruvate sulfurtransferase	Transfers sulfur from mercaptopyruvate to form thiocyanate, cleaving a carbon–sulfur bond (unlike rhodanese)	Vertebrates, plants, protozoa and bacteria	[23,26]
cystathionine λ-lyase	Transfers sulfur between cysteine molecules generating thiocysteine and pyruvate; produces thiosulfate for rhodanese pathway	Mammals	[26]
Hydrolytic reactions			
cyanide hydratase	Converts hydrogen cyanide to formamide	Fungi	[21,25]
nitrilase	Converts nitriles directly to carboxylic acids and ammonia; hydrolyzes toxic β-cyanoalanine to non-toxic asparagine/aspartate	Plants, fungi, certain protozoa, nematodes, insects	[21,25,27]
nitrile hydratase	Degrades nitriles to corresponding amides	Bacteria, fungi and yeasts	[21,25]
cyanidase	Directly converts cyanide to formate and ammonia	Bacteria	[25]
Oxidative reaction			
cyanide oxygenases	Two classes: monooxygenase converts cyanide to cyanate; dioxygenase directly oxidizes cyanide to carbon dioxide and ammonia	Bacteria and fungi	[25]
Cyanide-resistant respiration			
mitochondrial alternative oxidase	Transfers electrons from ubiquinol to oxygen, bypassing complex IV of MRC and creating cyanide-insensitive pathway	Plants, fungi, protists, invertebrates	[28,29,30,31]

**Table 2:** Protein sequences identified in available tardigrade genomic data. Record marked with asterisk (*) contains an erroneous large fragment, appended to the C-terminus [30,42].

Tardigrade Species	Encoded Protein	Genbank ID	Citation
Alternative oxidase			
*Hys. exemplaris*	Alternative oxidase	OWA52662.1 *	[73]
*Pam. metropolitanus*	Alternative oxidase	XP_055347198.1	unpublished
*Ram. varieornatus*	Alternative oxidase	GAU99582.1	[74]
Nitrilase superfamily			
*Hys. exemplaris*	Beta-ureidopropionase (UPB1)	OWA50527.1	[73]
	Carbon–nitrogen hydrolase	OWA52515.1	[73]
	Nitrilase and fragile histidine triad fusion protein NitFhit	OQV16277.1	[73]
	Nitrilase and fragile histidine triad fusion protein NitFhit	OQV23990.1	[73]
	Pantetheinase	OQV16046.1	[73]
*Pam. metropolitanus*	Beta-ureidopropionase (UPB1)	XP_055327677.1	unpublished
	Nitrilase and fragile histidine triad fusion protein NitFhit	XP_055334227.1	unpublished
	Nitrilase and fragile histidine triad fusion protein NitFhit	XP_055334234.1	unpublished
	Nitrilase and fragile histidine triad fusion protein NitFhit	XP_055334241.1	unpublished
	Nitrilase and fragile histidine triad fusion protein NitFhit	XP_055353777.1	unpublished
	Pantetheinase	XP_055327946.1	unpublished
*Ram. varieornatus*	Beta-ureidopropionase (UPB1)	GAV03454.1	[74]
	Nitrilase and fragile histidine triad fusion protein NitFhit	GAU93357.1	[74]
	Nitrilase and fragile histidine triad fusion protein NitFhit	GAV03569.1	[74]
	Nitrilase and fragile histidine triad fusion protein NitFhit	GAV03570.1	[74]
	Pantetheinase	GAV09019.1	[74]
Rhodanese superfamily			
*Hys. exemplaris*	Thiosulfate sulfurtransferase-like	OQV11887.1	[73]
	Thiosulfate sulfurtransferase-like	OQV11889.1	[73]
*Pam. metropolitanus*	Uncharacterized rhodanese superfamily protein	XP_055348691.1	unpublished
	Uncharacterized rhodanese superfamily protein	XP_055348693.1	unpublished
	Uncharacterized rhodanese superfamily protein	XP_055348692.1	unpublished
	Thiosulfate sulfurtransferase-like	XP_055342607.1	unpublished
	Thiosulfate: glutathione sulfurtransferase	XP_055328459.1	unpublished
	Thiosulfate: glutathione sulfurtransferase	XP_055328460.1	unpublished
*Ram. varieornatus*	Hypothetical protein (3-mercaptopyruvate sulfurtransferase SseA, contains two rhodanese domains)	GAU96454.1	[74]

**Table 3:** Mean recovery time of *Hys. exemplaris* after exposure to 100 mM KCN (*n* = 30 for each group), compared with recovery times for *Pam. experimentalis* and *Mac. naginae* subjected to the same conditions, according to Bartylak et al. [46].

Tardigrade Species	Mean Recovery Time [Min ± SD]	Source of Data
*Hys. exemplaris*	19.00 ± 4.24	Current study
*Pam. experimentalis*	20.00 ± 7.88	[46]
*Mac. naginae*	19.33 ± 7.04	[46]

**Table 4:** Distribution of genes encoding cyanide-resistance-associated proteins across analyzed eutardigrade genomes. X indicates presence of the sequence, while - indicates its absence.

Protein		Tardigrade Species		
		*Hys. exe.*	*Pam. met.*	*Ram. var.*
Alternative oxidase		X	X	X
Nitrilase superfamily	Beta-ureidopropionase (UPB1)	X	X	X
	Nitrilase and fragile histidine triad fusion protein NitFhit	X	X	X
	Pantetheinase	X	X	X
	Carbon–nitrogen hydrolase	X	-	-
Rhodanese superfamily	Thiosulfate sulfurtransferase-like	X	X	-
	Uncharacterized rhodanese superfamily protein	-	X	-
	Thiosulfate: glutathione sulfurtransferase	-	X	-
	Hypothetical protein (3-mercaptopyruvate sulfurtransferase SseA, contains two rhodanese domains)	-	-	X

## Data Availability

The datasets used and/or analysed during the current study are available from the corresponding author on reasonable request.

## Supplementary Materials

The following supporting information can be downloaded at https://www.mdpi.com/article/10.3390/ijms27114946/s1.
