# Supplementary material for: Candidate Cyanide Resistance Genes in Eutardigrade (Tardigrada) Genomes and KCN Resistance of Hypsibius exemplaris
Source: Int J Mol Sci. 2026 May 29;27(11):4946. doi: 10.3390/ijms27114946 (PMC13257105; doi:10.3390/ijms27114946)
Supplement: Supplementary file 1 [file ijms-27-04946-s001.zip › Figure S1_Nitrilase_tree_bootstrap.pdf]

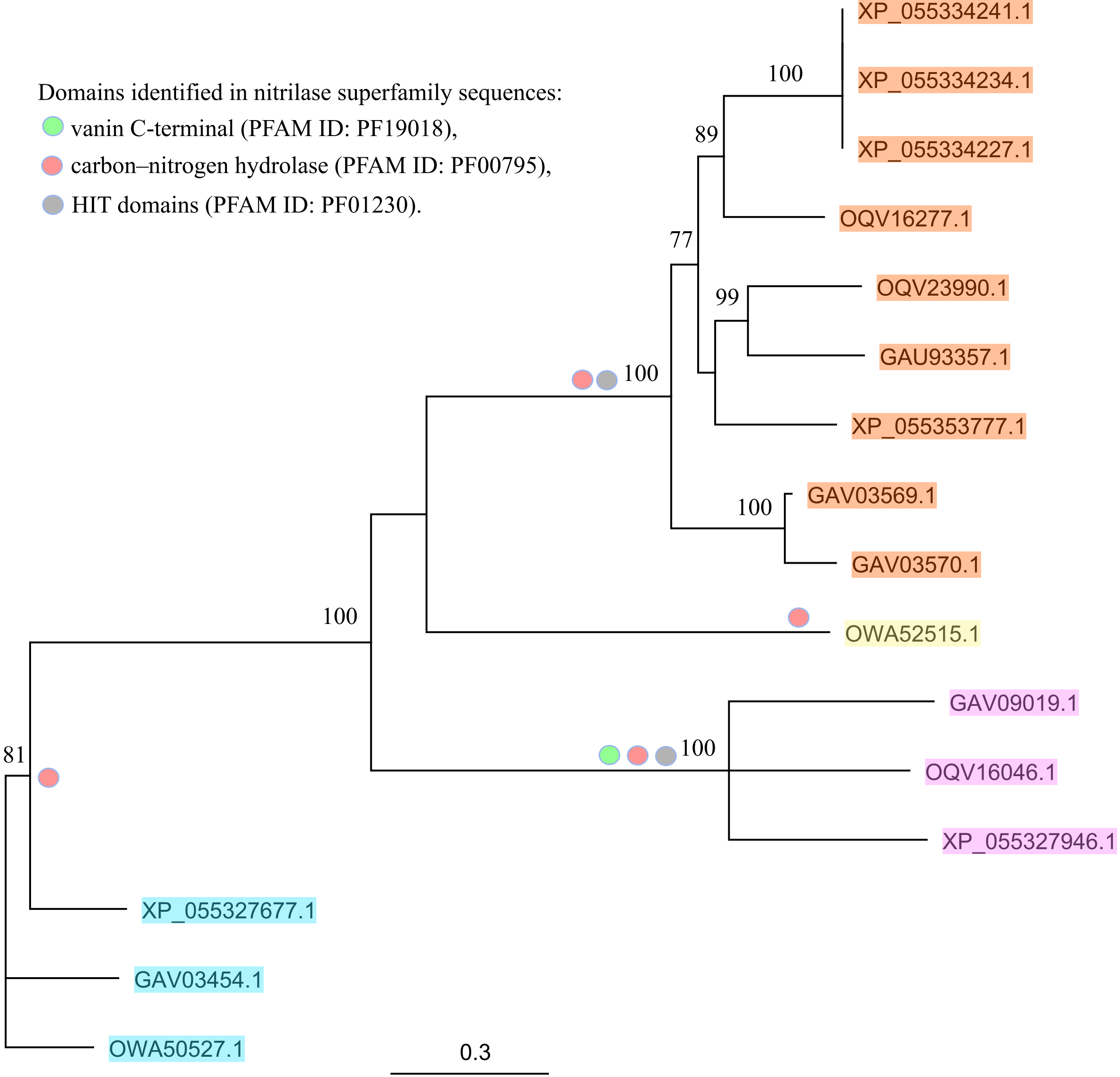

Domains identified in nitrilase superfamily sequences:
vanin C-terminal (PFAM ID: PF19018),
carbon–nitrogen hydrolase (PFAM ID: PF00795),
HIT domains (PFAM ID: PF01230).
XP_055334241.1
XP_055334234.1
XP_055334227.1
OQV16277.1
OQV23990.1
GAU93357.1
XP_055353777.1
GAV03569.1
GAV03570.1
OWA52515.1
GAV09019.1
OQV16046.1
XP_055327946.1
XP_055327677.1
GAV03454.1
OWA50527.1
100
89
77
99
100
100
100
100
81
0.3
