# Supplementary material for: Candidate Cyanide Resistance Genes in Eutardigrade (Tardigrada) Genomes and KCN Resistance of Hypsibius exemplaris
Source: Int J Mol Sci. 2026 May 29;27(11):4946. doi: 10.3390/ijms27114946 (PMC13257105; doi:10.3390/ijms27114946)
Supplement: Supplementary file 1 [file ijms-27-04946-s001.zip › SM Captions.pdf]

Supplementary Materials Captions

Table S1 Pairwise amino acid sequence identity (%) of nitrilase proteins identified in analyzed tardigrade genomes.

Table S2 Pairwise amino acid sequence identity (%) of rhodanese proteins identified in analyzed tardigrade genomes.

Figure S1 Neighbor-Joining consensus tree showing the relationships among nitrilase amino acid sequences identified in the analyzed tardigrade genomes. The topology was inferred from a multiple sequence alignment of the analyzed protein sequences. Bootstrap values are shown on the branches of the dendrogram; different nitrilase homologs are indicated by distinct colors; and identified domains within sequences belonging to the nitrilase superfamily are indicated.

Figure S2 Neighbor-Joining consensus tree showing the relationships among rhodanese amino acid sequences identified in the analyzed tardigrade genomes. The topology was inferred from a multiple sequence alignment of the analyzed protein sequences. Bootstrap values are shown on the branches of the dendrogram and different rhodanese homologs are indicated by distinct colors.
