# Supplementary figures and images for: Candidate Cyanide Resistance Genes in Eutardigrade (Tardigrada) Genomes and KCN Resistance of Hypsibius exemplaris
Source: Int J Mol Sci. 2026 May 29;27(11):4946. doi: 10.3390/ijms27114946 (PMC13257105; doi:10.3390/ijms27114946)

A rhodanese-like domain (PFAM ID: PF00581)  
was identified in all rhodanese family sequences.

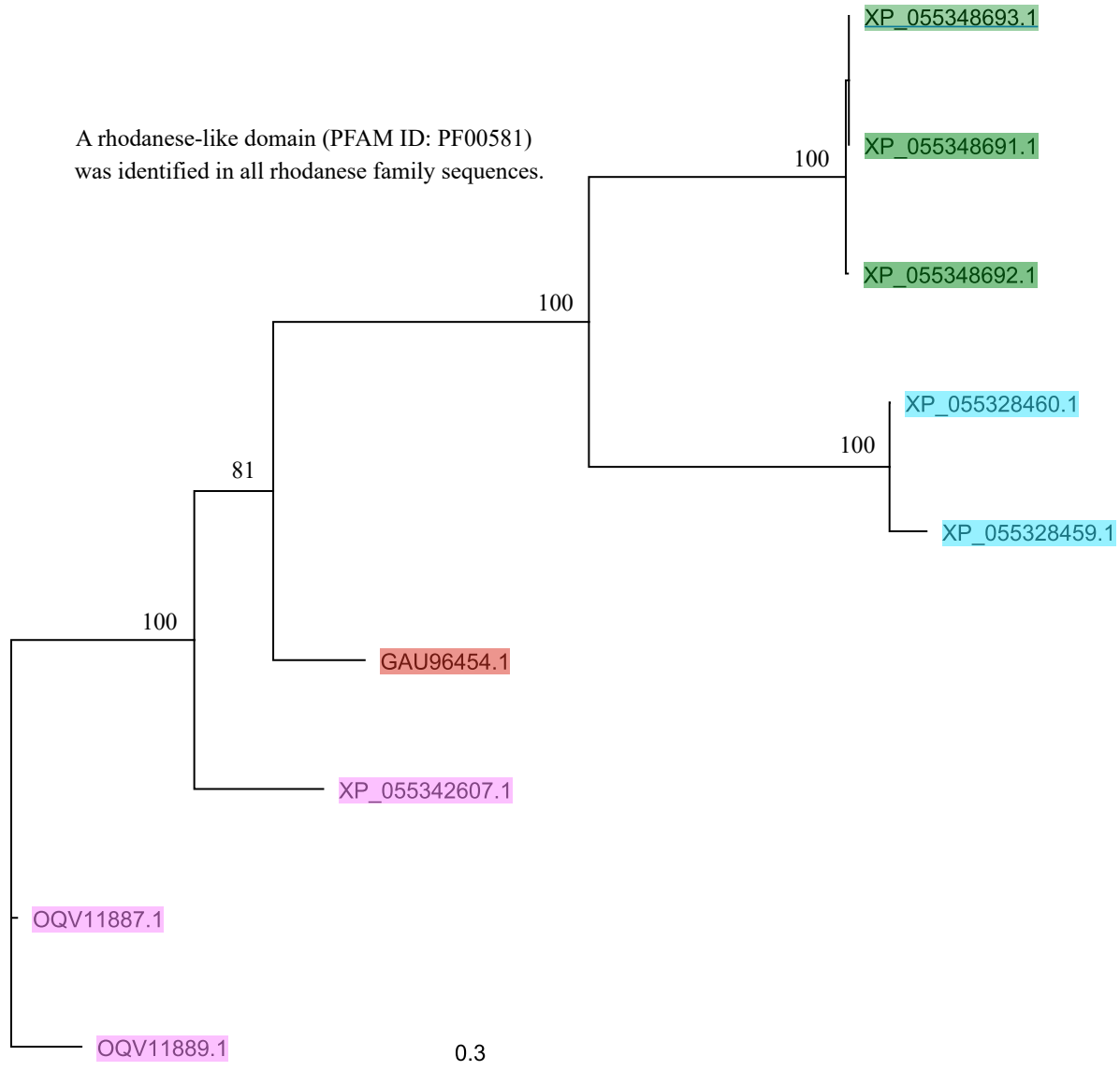

Supplement: Supplementary file 1 [file ijms-27-04946-s001.zip › Figure S2_Rhodonase_tree_bootstrap.pdf]
